# Supplementary material for: The seminal odorant binding protein Obp56g is required for mating plug formation and male fertility in Drosophila melanogaster
Source: bioRxiv. 2023 Feb 7:2023.02.03.526941. Preprint. [Version 1] doi: 10.1101/2023.02.03.526941 (PMC9934574; doi:10.1101/2023.02.03.526941)
Supplement: Supplement 1 [file NIHPP2023.02.03.526941v1-supplement-1.pdf]

ioRxiv preprint doi: https://doi.org/10.1101/2023.02.03.526941; this version posted February 7, 2023. The copyright holder for this preprint
hich was not certified by peer review) is the author/funder, who has granted bioRxiv a license to display the preprint in perpetuity. It is made
available under aCC-BY 4.0 International license.

Supplemental figures & figure legends:

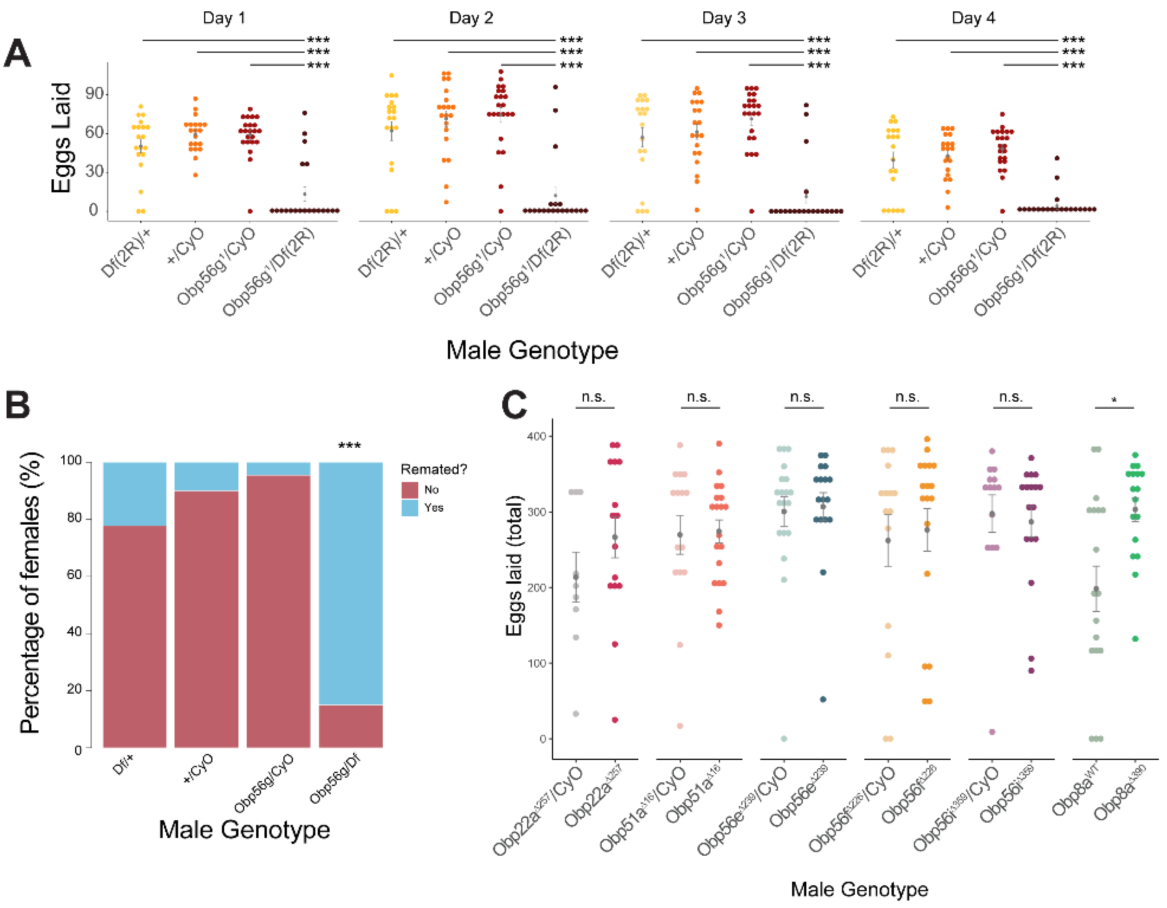

Figure 1—figure supplement 1: Additional replicate of PMR phenotypes from CS females mated to *Obp56g* and CRISPR mutant males. A) Egg counts from CS females mated *to Df(2R)/+, CyO/+ Obp56g^{1}/CyO*, or *Obp56g^{1}/Df(2R)* males from 1-4 days after mating. Significance indicated from pairwise comparisons of male genotypes within days using emmeans on a Poisson linear mixed effects model. Error bars represent mean +/- SEM. B) Proportion of females who did or did not remate with a standard CS male on the fourth day after mating. Significance indicated from tests of equality of proportions. C) Egg counts from CS females mated to homozygous null or heterozygous control males (except for *Obp8a*, the control of which is from an unedited sibling line) from 1-4 days after mating. Error bars represent mean +/- SEM. Significance indicated from Poisson linear models with Benjamini-Hochberg corrections for multiple comparisons. Significance levels: *P<0.05, **P<0.01, ***P<0.001, n.s. not significant.

bioRxiv preprint doi: https://doi.org/10.1101/2023.02.03.526941; this version posted February 7, 2023. The copyright holder for this preprint (which was not certified by peer review) is the author/funder, who has granted bioRxiv a license to display the preprint in perpetuity. It is made available under aCC-BY 4.0 International license.

Figure 1—figure supplement 1—source data 1: Remating counts and percentages for data shown in Figure 1—figure supplement 1B.

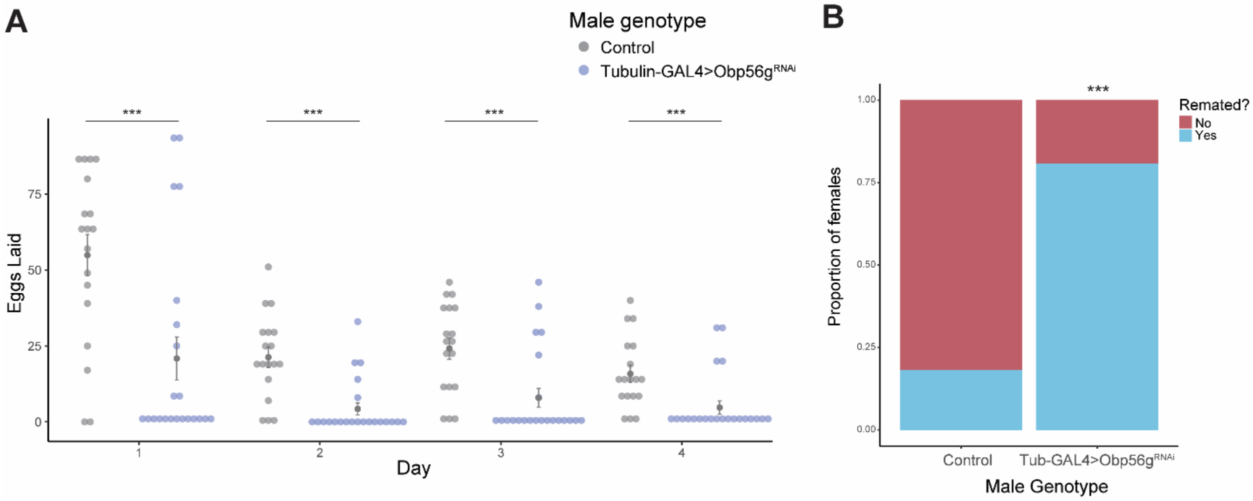

Figure 1—figure supplement 2: Whole body knockdown of *Obp56g* using *Tubulin-GAL4* results in loss of post-mating response phenotypes in females. (A) Counts of eggs from mated CS females over 4 days. Females mated to *Tubulin-GAL4>Obp56g*^{RNAi} males lay significantly fewer eggs than females mated to control males (p<0.001, n=20-24). (B) CS females mated to *Tubulin-GAL4>Obp56g*^{RNAi} males are significantly more likely to remate 4 days post-mating relative to control males (p<0.001, n=26-33). Error bars represent mean +/- SEM. Significance level: * p<0.05, ** p<0.01, *** p<0.001.

Figure 1—figure supplement 2—source data 1: Remating counts and percentages for data shown in Figure 1—figure supplement 2B.

bioRxiv preprint doi: https://doi.org/10.1101/2023.02.03.526941; this version posted February 7, 2023. The copyright holder for this preprint (which was not certified by peer review) is the author/funder, who has granted bioRxiv a license to display the preprint in perpetuity. It is made available under aCC-BY 4.0 International license.

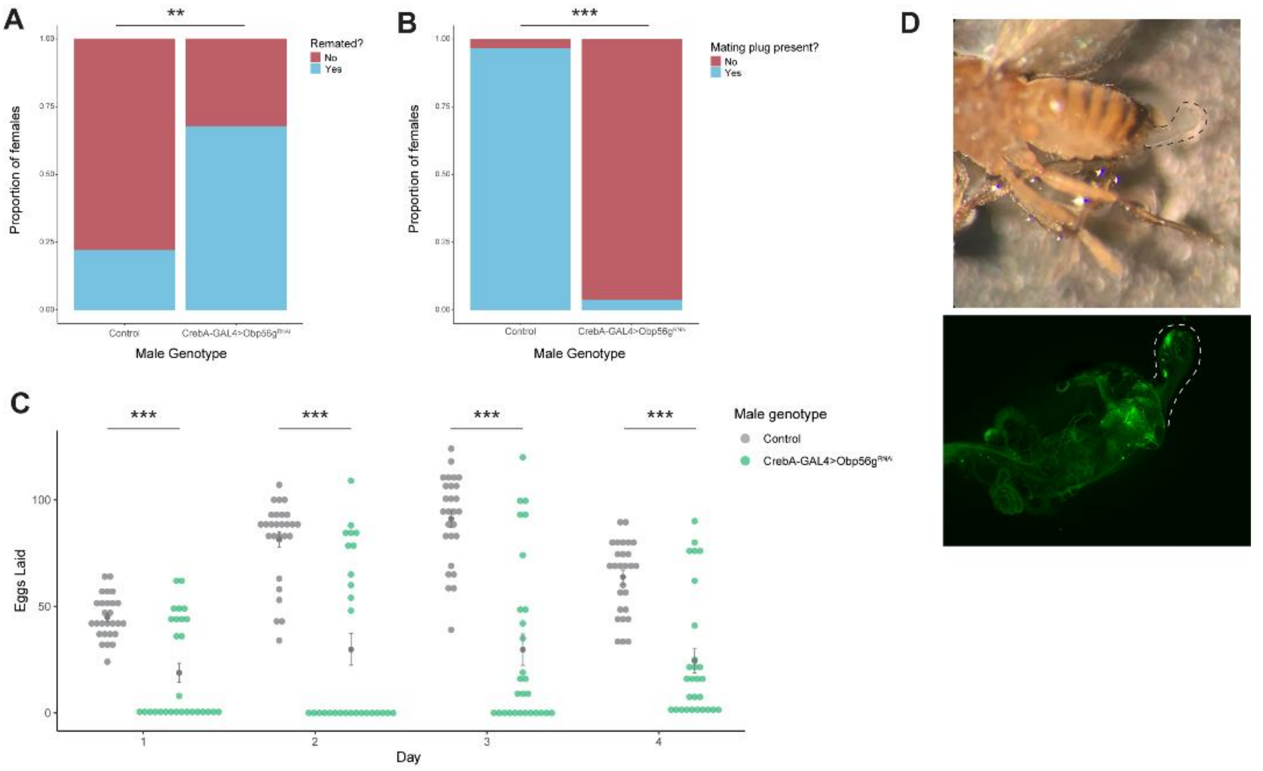

Figure 1—figure supplement 3: Male reproductive tract knockdown of *Obp56g* with *CrebA-GAL4* is required for the post-mating response and mating plug formation. (A) CS females mated to *CrebA-GAL4>Obp56g*^{RNAi} males are significantly more likely to remate 4 days post-mating relative to control males (p=0.001, n=27-28). (B) A significantly reduced proportion of females mated to *CrebA-GAL4>Obp56g*^{RNAi} males have fully formed mating plugs in their bursa immediately after the end of mating relative to females mated to control males (p<0.001, n=27-30). (C) Counts of eggs from mated CS females over 4 days. CS females mated to *CrebA-GAL4>Obp56g*^{RNAi} lay significantly fewer numbers of eggs relative to CS females mated to control males (p<0.001, n=27-28). (D) Ejaculate loss (dotted line) from the bursa observed in females mated to *CrebA-GAL4>Obp56g*^{RNAi} males, with the bursa dissected and imaged for GFP to visualize the autofluorescent speckles that comprise the uncoagulated mating plug. Error bars represent mean +/- SEM. Significance level: * p<0.05, ** p<0.01, *** p<0.001.

Figure 1—figure supplement 3—source data 1: Counts and percentages for data shown in Figure 1—figure supplement 3A & B.

bioRxiv preprint doi: https://doi.org/10.1101/2023.02.03.526941; this version posted February 7, 2023. The copyright holder for this preprint (which was not certified by peer review) is the author/funder, who has granted bioRxiv a license to display the preprint in perpetuity. It is made available under aCC-BY 4.0 International license.

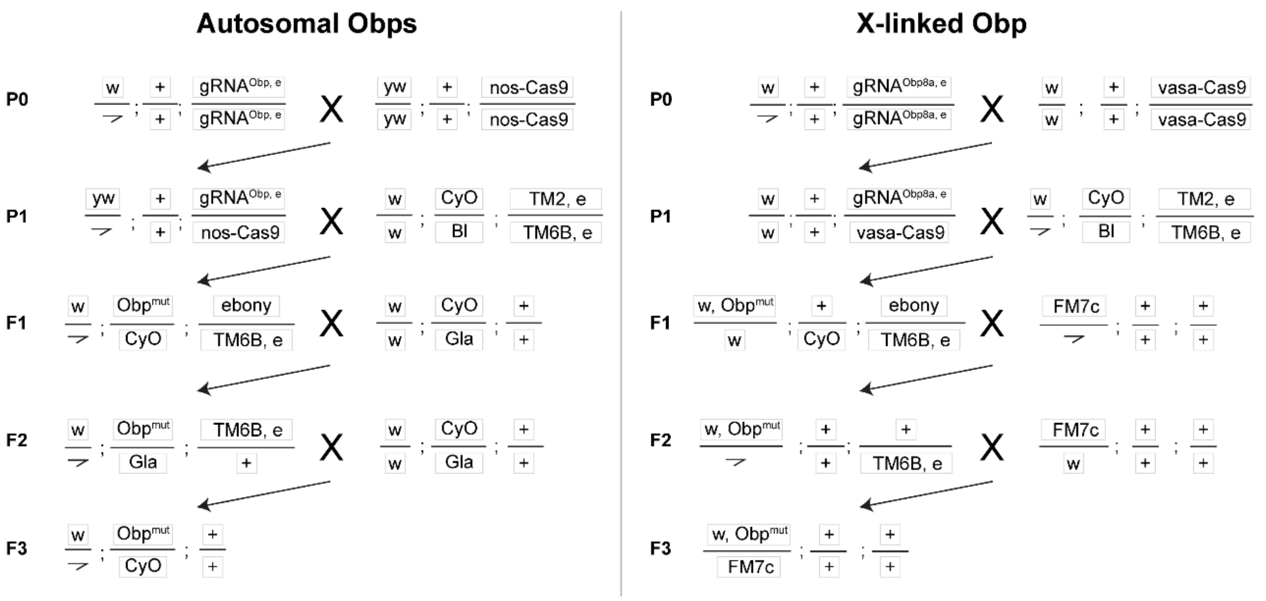

Figure 2—figure supplement 1: Crossing scheme to generate CRISPR mutants in autosomal (*Obp22a, Obp51a, Obp56e, Obp56f, Obp56i*) and X-linked (*Obp8a*) *Obp* genes used in this study, with text boxes representing chromosomes X/Y, 2, and 3 (dot chromosome not shown). The kinked line represents the Y chromosome. *Obp* and *ebony* CRISPR editing takes place in the germline of individuals in the P1 generation. *ebony* editing can happen on either the gRNA or Cas9 chromosomes (written out in the F1 generation as "*ebony*" for simplicity), which are removed from the genetic background before assaying males for reproductive phenotypes.

bioRxiv preprint doi: https://doi.org/10.1101/2023.02.03.526941; this version posted February 7, 2023. The copyright holder for this preprint (which was not certified by peer review) is the author/funder, who has granted bioRxiv a license to display the preprint in perpetuity. It is made available under aCC-BY 4.0 International license.

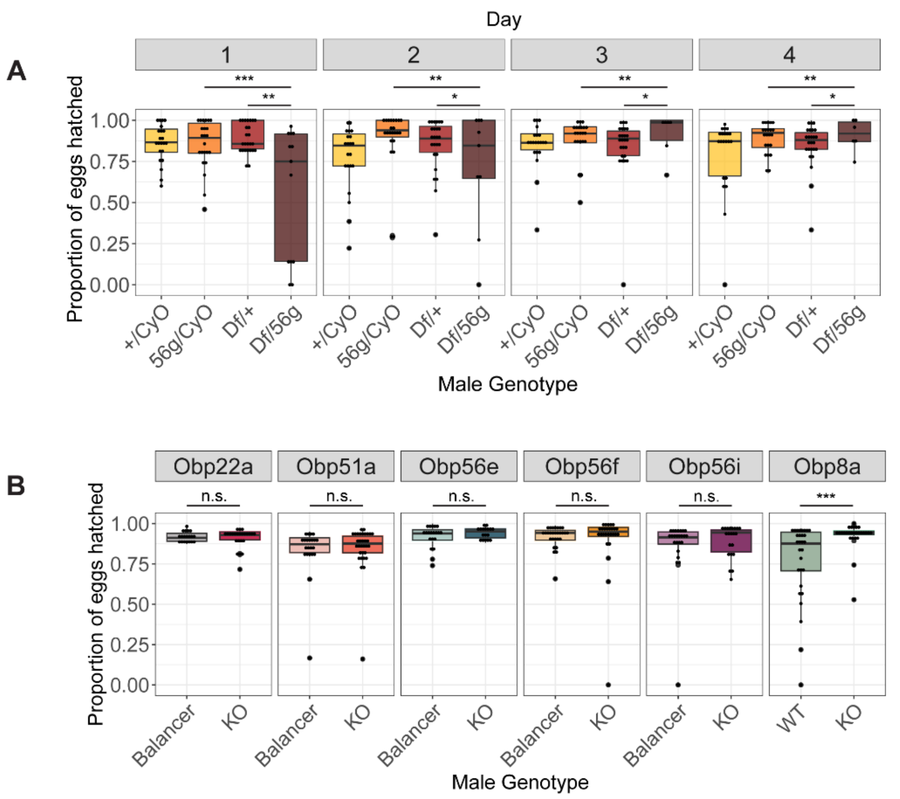

Figure 2—figure supplement 2: Box plots of hatchability estimates from CS females mated to *Obp56g* or CRISPR mutant males. A) Proportion of eggs hatched over 4 days from females mated to *Df(2R)/+, CyO/+ Obp56g*^{1}*/CyO*, or *Obp56g*^{1}*/Df(2R)* males. Significance indicated from pairwise comparisons of male genotypes across days using emmeans on a binomial mixed effects model. B) Proportion of eggs hatched over 4 days from females mated to CRISPR mutant males. Significance indicated from binomial linear models with Benjamini-Hochberg corrections for multiple comparisons. Significance levels: *P<0.05, **P<0.01, ***P<0.001, n.s. not significant.

bioRxiv preprint doi: https://doi.org/10.1101/2023.02.03.526941; this version posted February 7, 2023. The copyright holder for this preprint (which was not certified by peer review) is the author/funder, who has granted bioRxiv a license to display the preprint in perpetuity. It is made available under aCC-BY 4.0 International license.

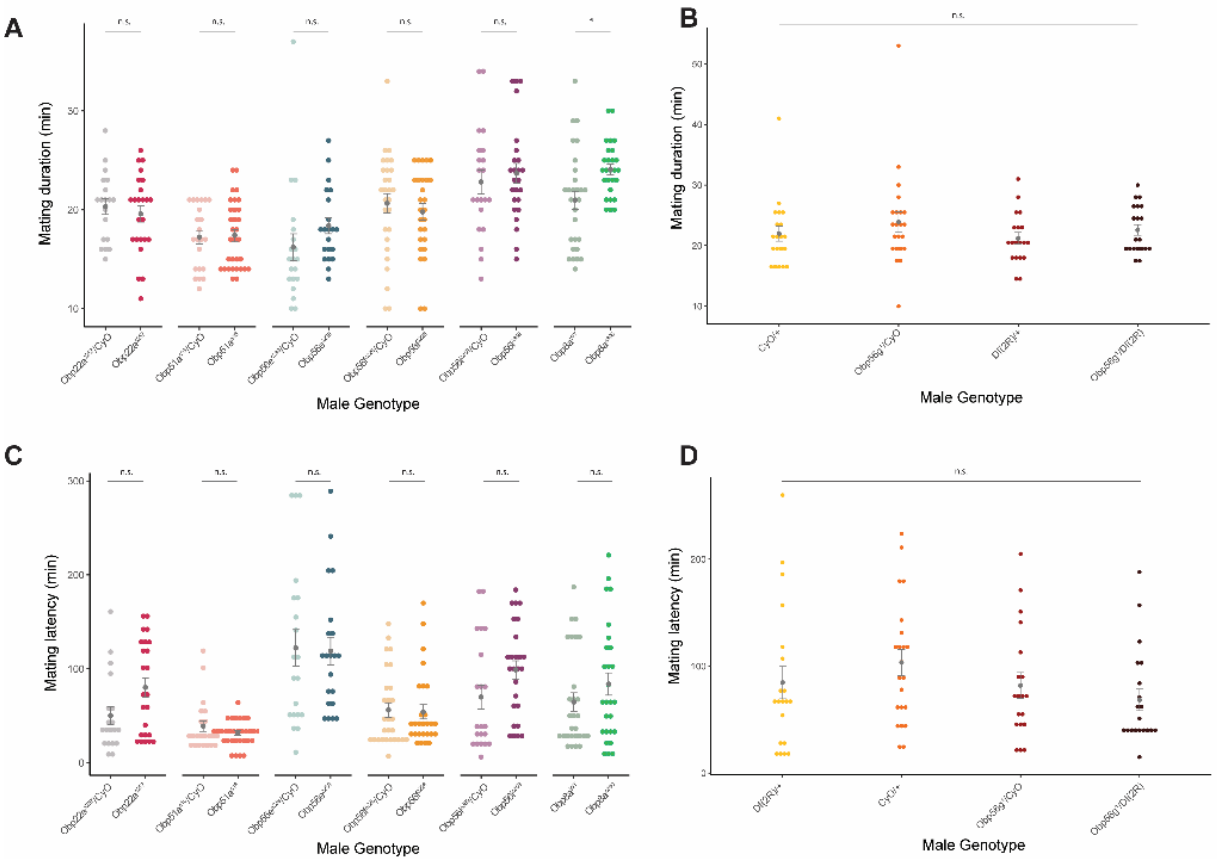

Figure 2—figure supplement 3: Mating latency and duration measurements from *Obp56g*^{1} and CRISPR-generated *Obp* mutants with CS females. (A & B) *Obp8a*$^{\Delta390}$ mutant flies mate for longer duration than *Obp8a*^{WT} control flies (p<0.05, mean *Obp8a*^{WT} 20.96 minutes, mean *Obp8a*$^{\Delta390}$ 24.07 minutes), though no other statistically significant differences were observed between mating duration of mutant or control males for other genotypes (p>0.05 for ANOVA [*Obp56g*^{1}] or Benjamini-Hochberg corrected p-values from Student's t-tests [CRISPR mutants]). (C & D) No statistically significant differences observed between mating latency of mutant or control males with CS females (p>0.05 for ANOVA [*Obp56g*^{1}] or Benjamini-Hochberg corrected p-values from Student's t-tests [CRISPR mutants]). *n* for each genotype ranged from 19 to 32. Error bars represent mean +/- SEM. Significance level: * p<0.05, ** p<0.01, *** p<0.001, n.s. not significant.

bioRxiv preprint doi: https://doi.org/10.1101/2023.02.03.526941; this version posted February 7, 2023. The copyright holder for this preprint (which was not certified by peer review) is the author/funder, who has granted bioRxiv a license to display the preprint in perpetuity. It is made available under aCC-BY 4.0 International license.

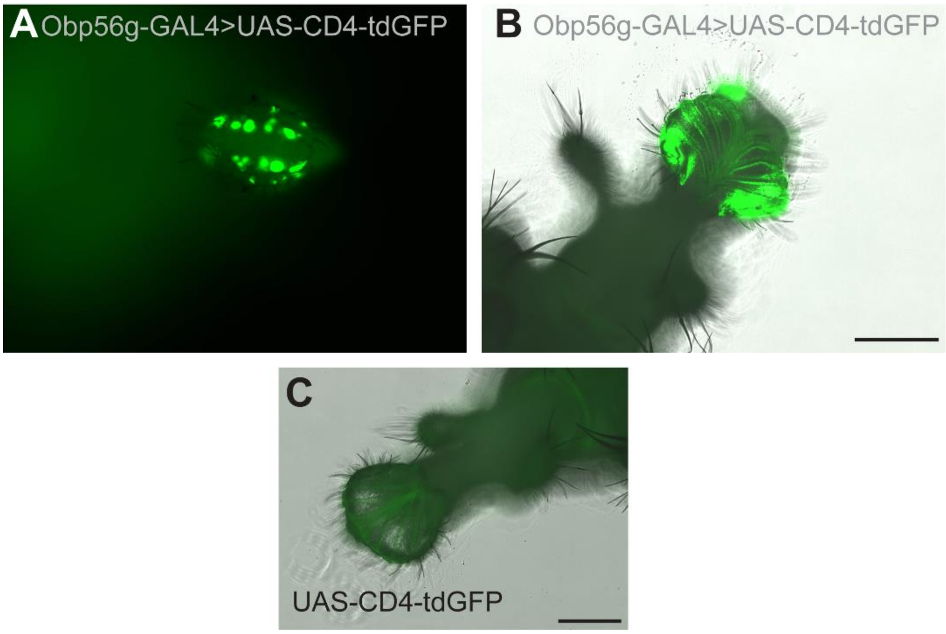

Figure 3—figure supplement 1: Expression of *Obp56g-GAL4* in the gustatory bristles of the labellum. A) GFP expression from *Obp56g-GAL>UAS-CD4-tdGFP* males in the head. This sample is not placed under a coverslip. B) GFP expression in the same genotype, with the proboscis dissected off and gently pressed under a coverslip. C) GFP expression in *UAS-CD4-tdGFP* control male labellum. Scale bar=130um.

bioRxiv preprint doi: https://doi.org/10.1101/2023.02.03.526941; this version posted February 7, 2023. The copyright holder for this preprint (which was not certified by peer review) is the author/funder, who has granted bioRxiv a license to display the preprint in perpetuity. It is made available under aCC-BY 4.0 International license.

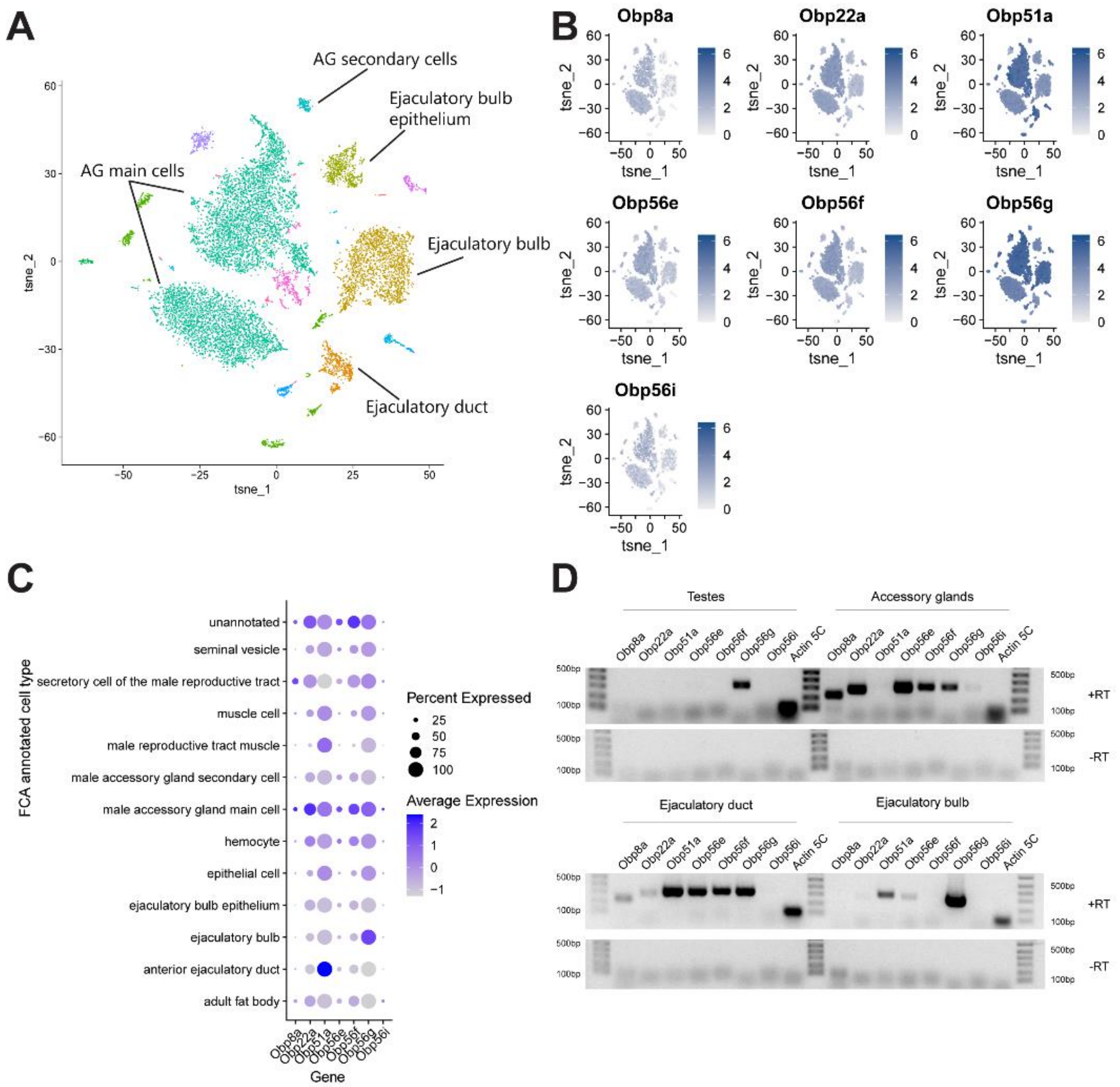

Figure 3—figure supplement 2: *Obp56g* is the most highly expressed seminal Obp in the ejaculatory bulb. A) Seurat tSNE dimensionality reduction plot of single nucleus RNAseq expression data from the male reproductive tract (without testes) and their major cell type annotations according to (Li et al., 2022). B) Feature plots from Seurat showing expression of the seminal *Obp* genes across single nuclei from A. C) Seurat dot plot of scaled average gene expression across annotated cell types for seminal Obps. Dot size indicates the percentage of cells within a cluster that express each *Obp* gene. D) Agarose gel of RT-PCR products of seminal *Obp* genes from microdissected bulk tissues of the *D. melanogaster* male reproductive tract (testes, accessory glands, ejaculatory ducts, and ejaculatory bulbs), with *Actin 5C* used as the positive control for each tissue. Samples treated with reverse transcriptase are above, and those without below (as a negative control), for each tissue type. PCR was performed for 35 cycles.

bioRxiv preprint doi: https://doi.org/10.1101/2023.02.03.526941; this version posted February 7, 2023. The copyright holder for this preprint (which was not certified by peer review) is the author/funder, who has granted bioRxiv a license to display the preprint in perpetuity. It is made available under aCC-BY 4.0 International license.

Figure 3—figure supplement 2—source data 1: Raw and uncropped, labeled gel images for data shown in Figure 3—figure supplement 2D.

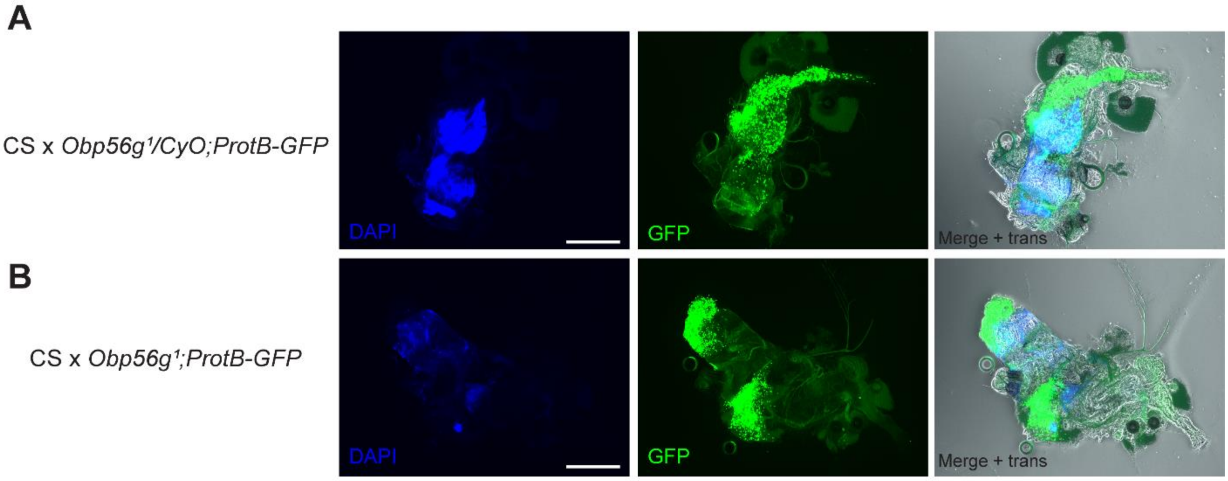

Figure 4—figure supplement 1: *Obp56g*^{1} mutant males do not have gross issues with sperm transfer during mating at the 12-minute ASM time point. A) Representative CS female mated to *Obp56g*^{1}*/CyO;ProtB-eGFP* control males, showing DAPI (mating plug), GFP (sperm heads), and merge + transillumination microscopy images. 9/9 females mated to these males had mating plugs, and 9/9 had sperm masses present in their bursas. B) Representative CS female mated to *Obp56g*^{1}*;ProtB-eGFP* mutant males. 0/10 females had mating plugs, though 10/10 had sperm masses present in their bursas.

bioRxiv preprint doi: https://doi.org/10.1101/2023.02.03.526941; this version posted February 7, 2023. The copyright holder for this preprint (which was not certified by peer review) is the author/funder, who has granted bioRxiv a license to display the preprint in perpetuity. It is made available under aCC-BY 4.0 International license.

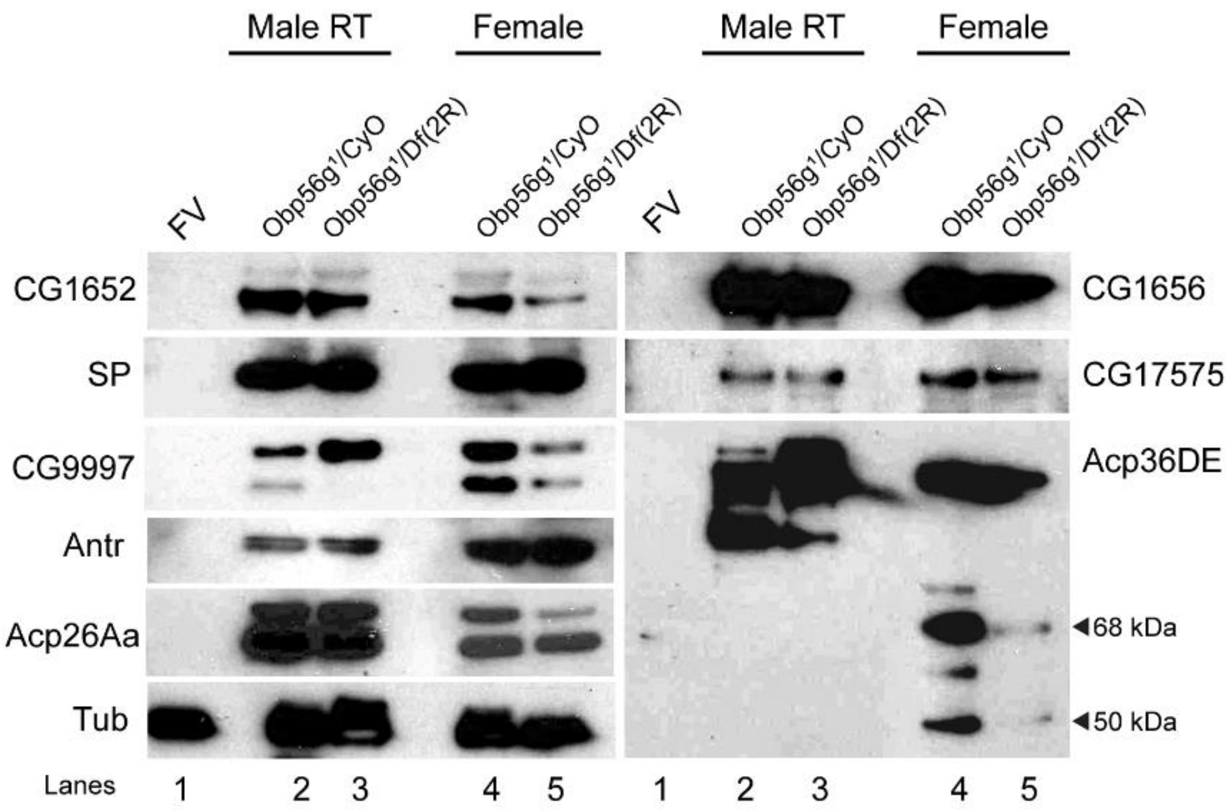

Figure 4—figure supplement 2: Females mated to *Obp56g*^{1} null males have reduced amounts of SFPs in their bursas 35 minutes ASM. Western blots for SFPs in 1) unmated female reproductive tracts, (2-3) male reproductive tracts, or (4-5) mated female reproductive tracts from CS females mated to either *Obp56g*^{1}*/CyO* control or *Obp56g*^{1}*/Df(2R)* males at 35 minutes ASM. All flies are 3-5 days old. Tubulin is shown as a loading control. Cleavage products of Acp36DE (68kDa and 50kDa) are shown with black arrows.

Figure 4—figure supplement 2—source data 1: Raw film images and uncropped, labeled Western blots for data shown in Figure 4—figure supplement 2.

bioRxiv preprint doi: https://doi.org/10.1101/2023.02.03.526941; this version posted February 7, 2023. The copyright holder for this preprint (which was not certified by peer review) is the author/funder, who has granted bioRxiv a license to display the preprint in perpetuity. It is made available under aCC-BY 4.0 International license.

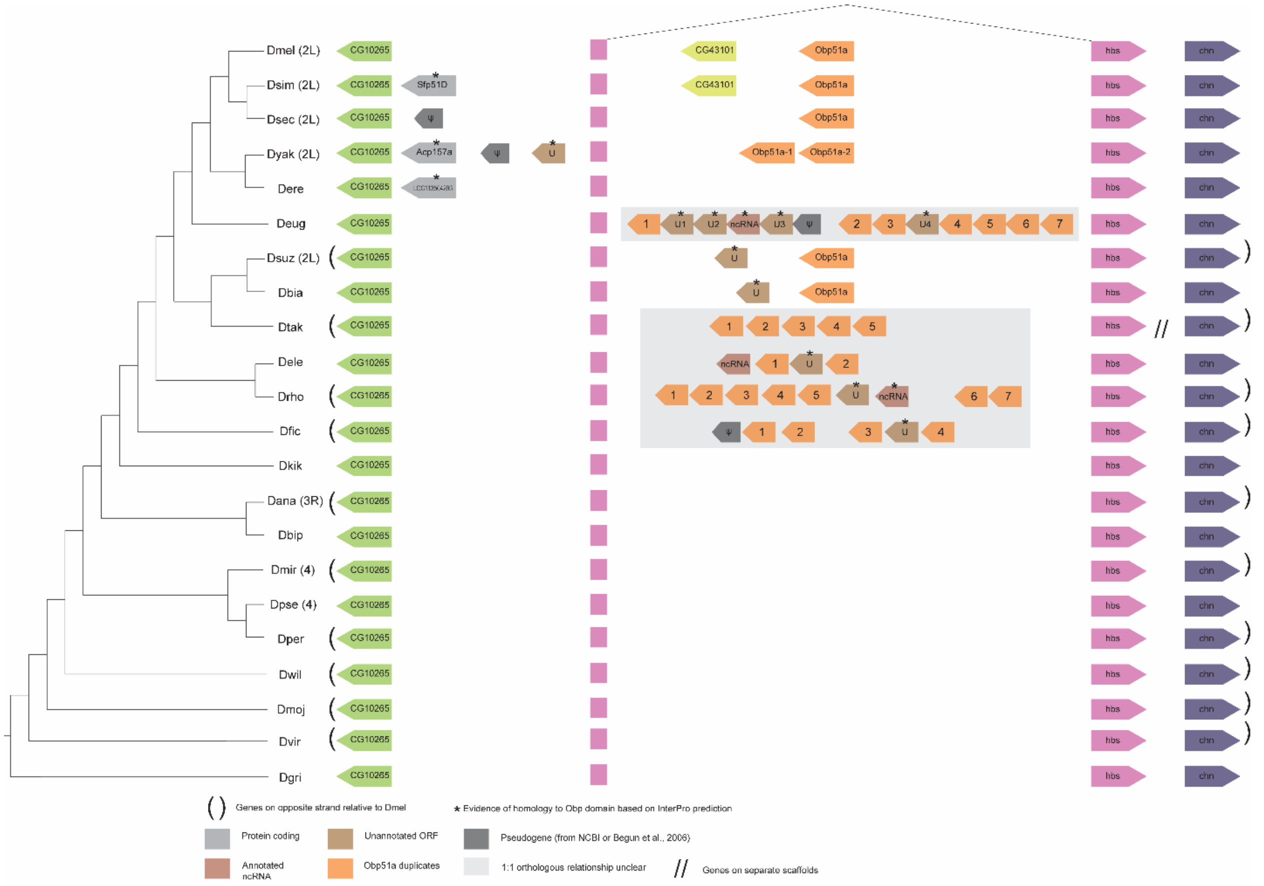

Figure 5—figure supplement 1: Synteny plot for *Obp51a*, phylogeny on the left from (McGeary and Findlay, 2020). Surrounding gene names represent gene names in *D. melanogaster*.

bioRxiv preprint doi: https://doi.org/10.1101/2023.02.03.526941; this version posted February 7, 2023. The copyright holder for this preprint (which was not certified by peer review) is the author/funder, who has granted bioRxiv a license to display the preprint in perpetuity. It is made available under aCC-BY 4.0 International license.

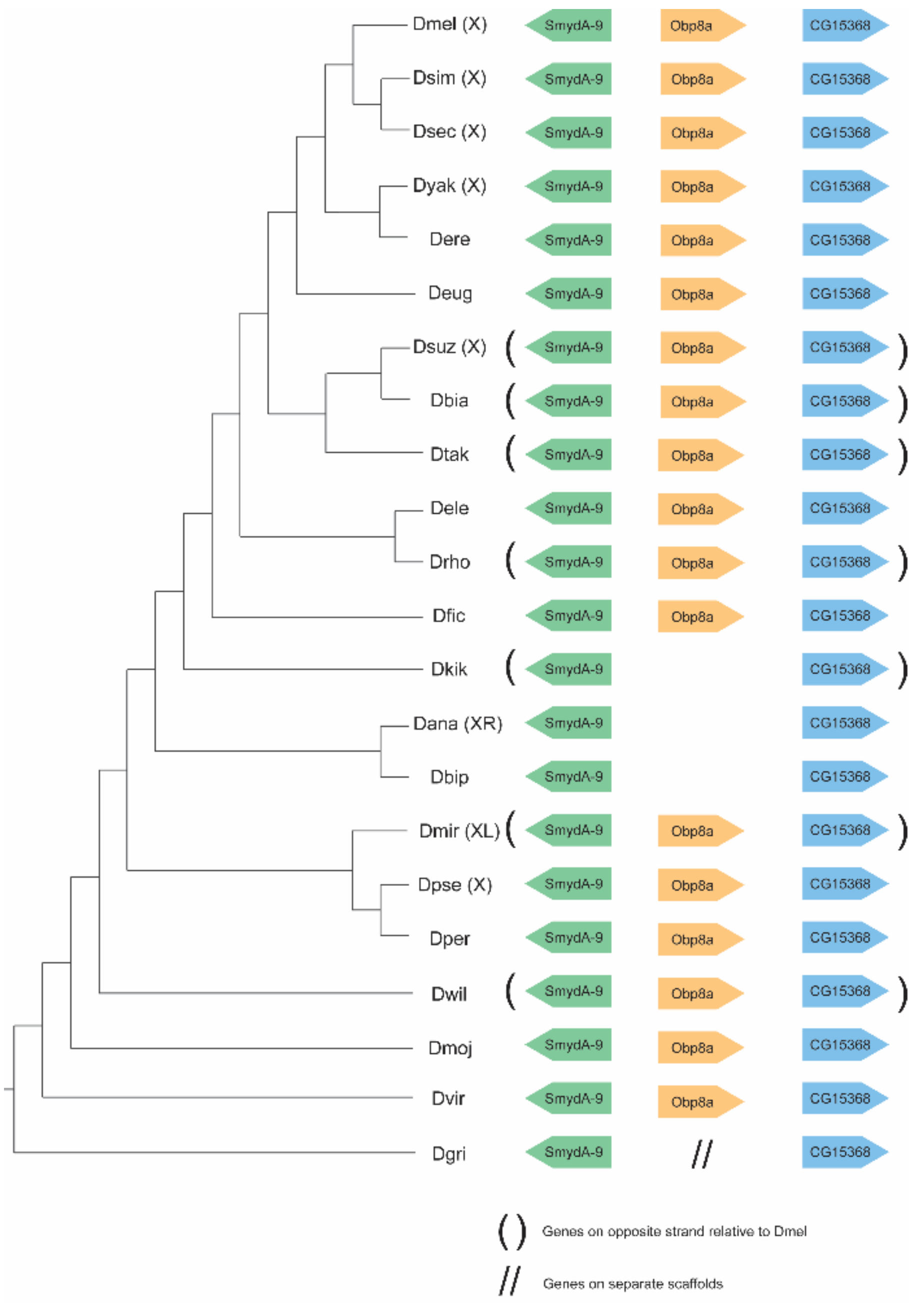

Figure 5—figure supplement 2: Synteny plot for *Obp8a*, phylogeny on the left from (McGeary and Findlay, 2020). Surrounding gene names represent gene names in *D. melanogaster*.

bioRxiv preprint doi: https://doi.org/10.1101/2023.02.03.526941; this version posted February 7, 2023. The copyright holder for this preprint (which was not certified by peer review) is the author/funder, who has granted bioRxiv a license to display the preprint in perpetuity. It is made available under aCC-BY 4.0 International license.

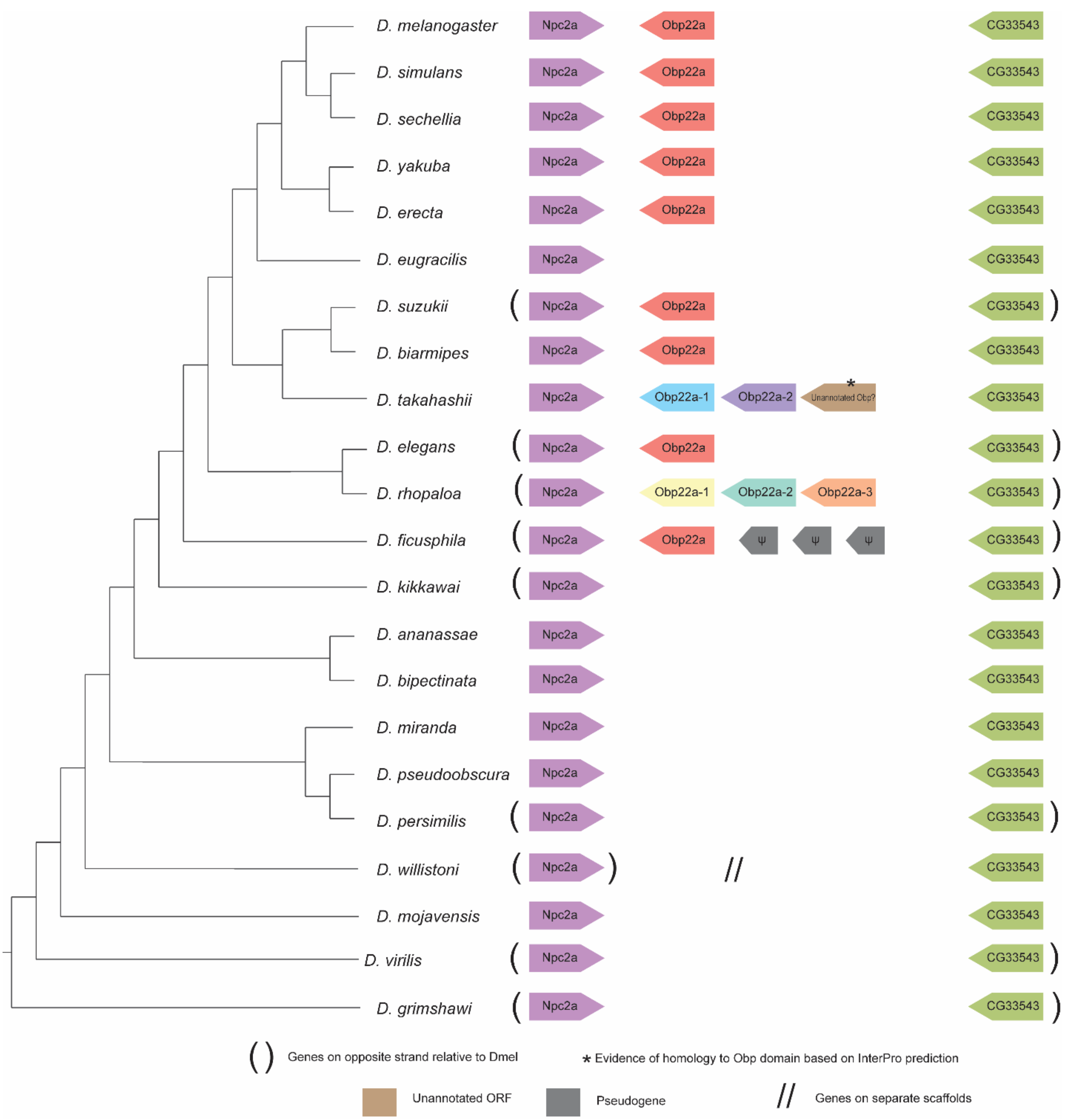

Figure 5—figure supplement 3: Synteny plot for *Obp22a*, phylogeny on the left from (McGeary and Findlay, 2020). Surrounding gene names represent gene names in *D. melanogaster*.

bioRxiv preprint doi: https://doi.org/10.1101/2023.02.03.526941; this version posted February 7, 2023. The copyright holder for this preprint (which was not certified by peer review) is the author/funder, who has granted bioRxiv a license to display the preprint in perpetuity. It is made available under aCC-BY 4.0 International license.

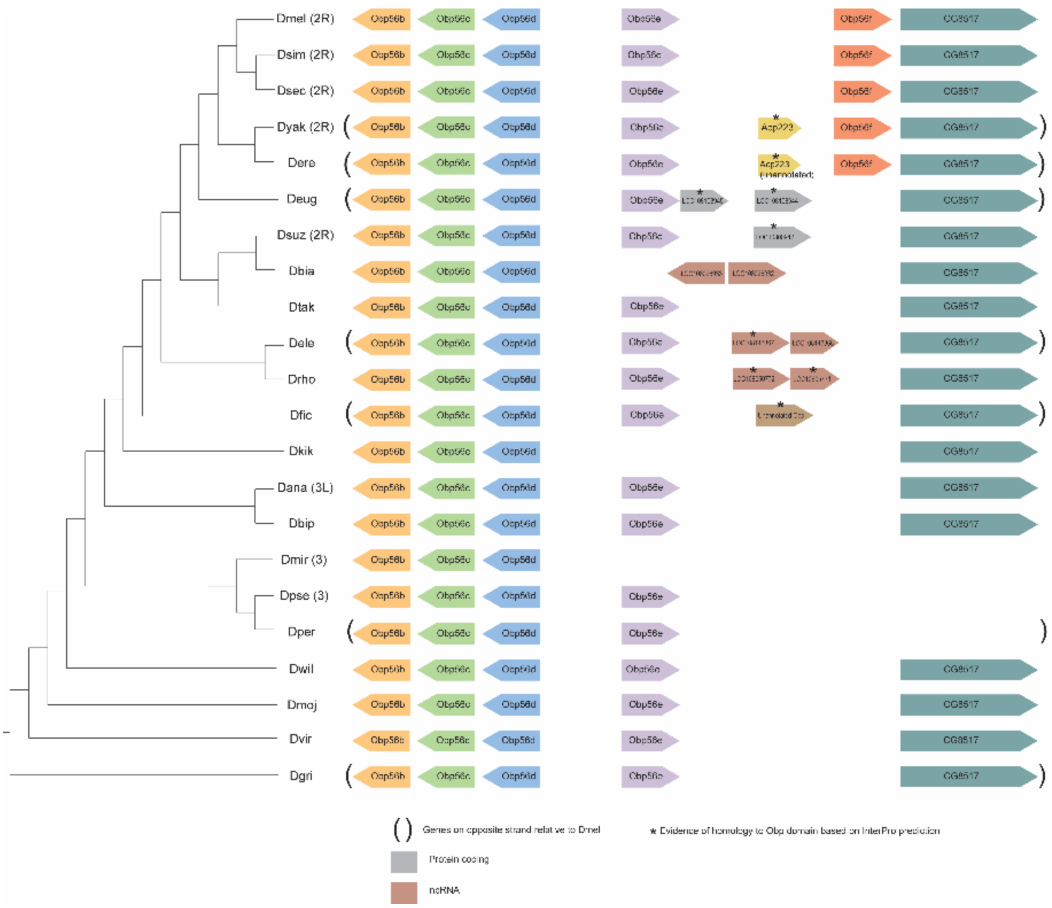

Figure 5—figure supplement 4: Synteny plot for *Obp56e* and *Obp56f*, phylogeny on the left from (McGeary and Findlay, 2020). Surrounding gene names represent gene names in *D. melanogaster*.

bioRxiv preprint doi: https://doi.org/10.1101/2023.02.03.526941; this version posted February 7, 2023. The copyright holder for this preprint (which was not certified by peer review) is the author/funder, who has granted bioRxiv a license to display the preprint in perpetuity. It is made available under aCC-BY 4.0 International license.

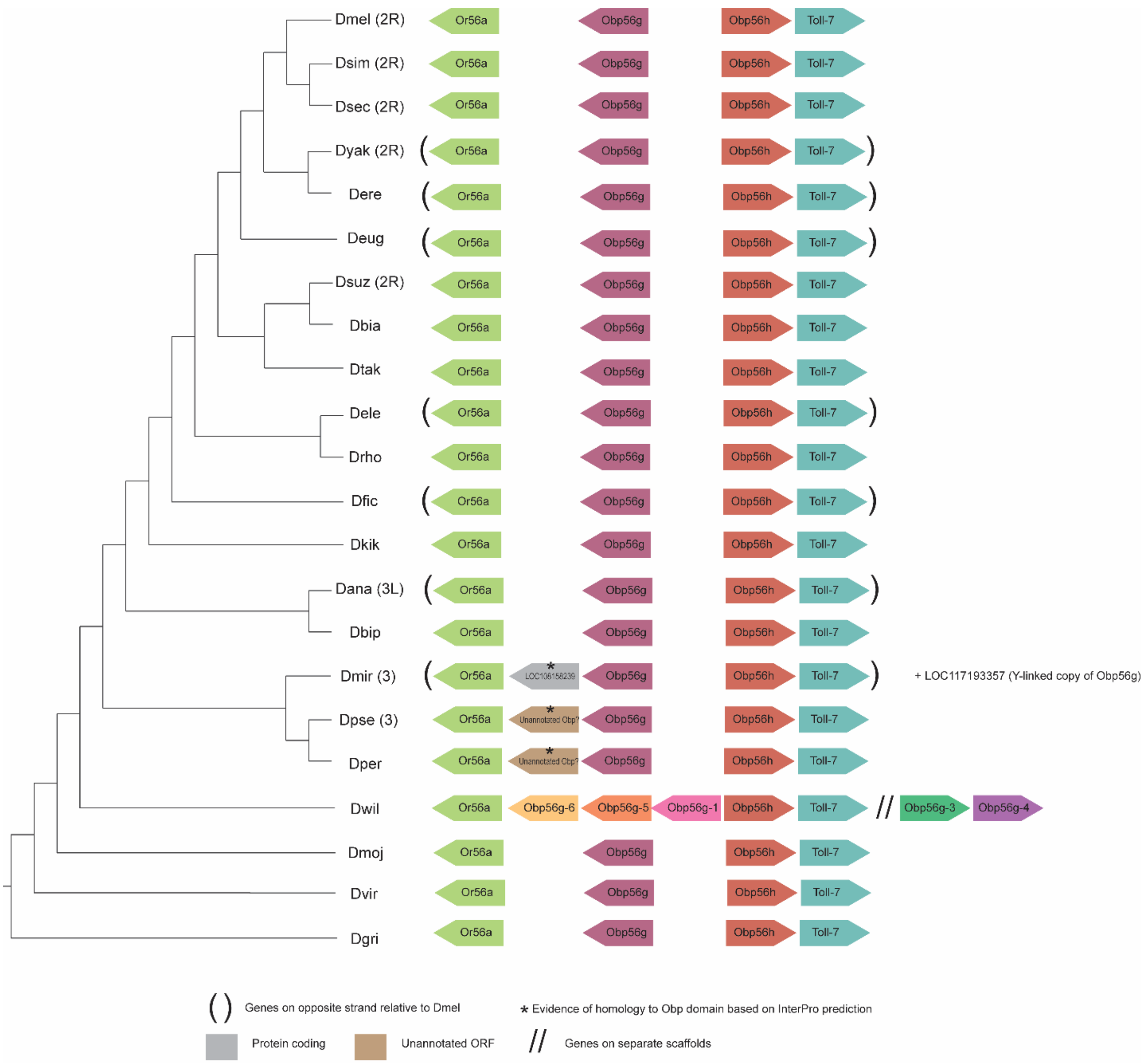

Figure 5—figure supplement 5: Synteny plot for *Obp56g*, phylogeny on the left from (McGeary and Findlay, 2020). Surrounding gene names represent gene names in *D. melanogaster*.

bioRxiv preprint doi: https://doi.org/10.1101/2023.02.03.526941; this version posted February 7, 2023. The copyright holder for this preprint (which was not certified by peer review) is the author/funder, who has granted bioRxiv a license to display the preprint in perpetuity. It is made available under aCC-BY 4.0 International license.

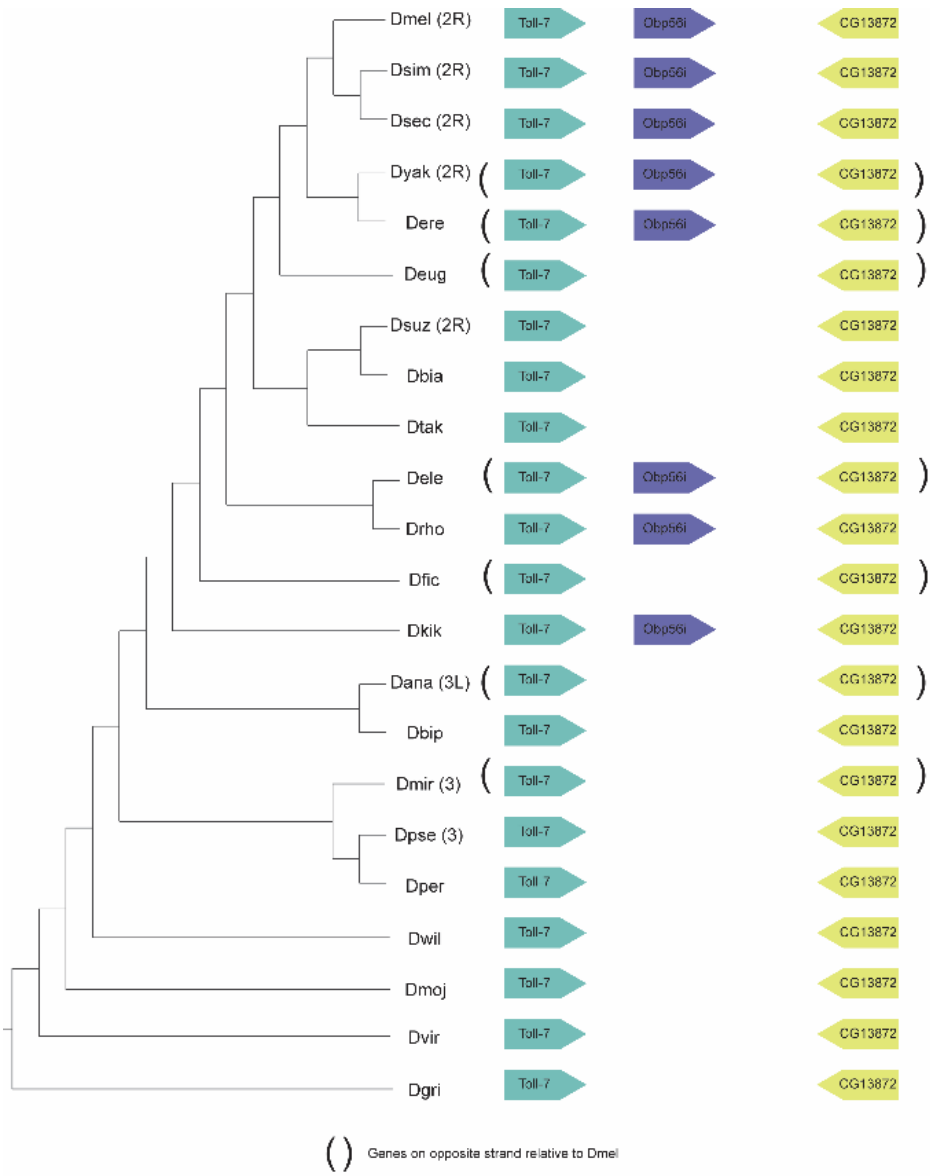

Figure 6—figure supplement 6: Synteny plot for *Obp56i*, phylogeny on the left from (McGeary and Findlay, 2020). Surrounding gene names represent gene names in *D. melanogaster*.

bioRxiv preprint doi: https://doi.org/10.1101/2023.02.03.526941; this version posted February 7, 2023. The copyright holder for this preprint (which was not certified by peer review) is the author/funder, who has granted bioRxiv a license to display the preprint in perpetuity. It is made available under aCC-BY 4.0 International license.

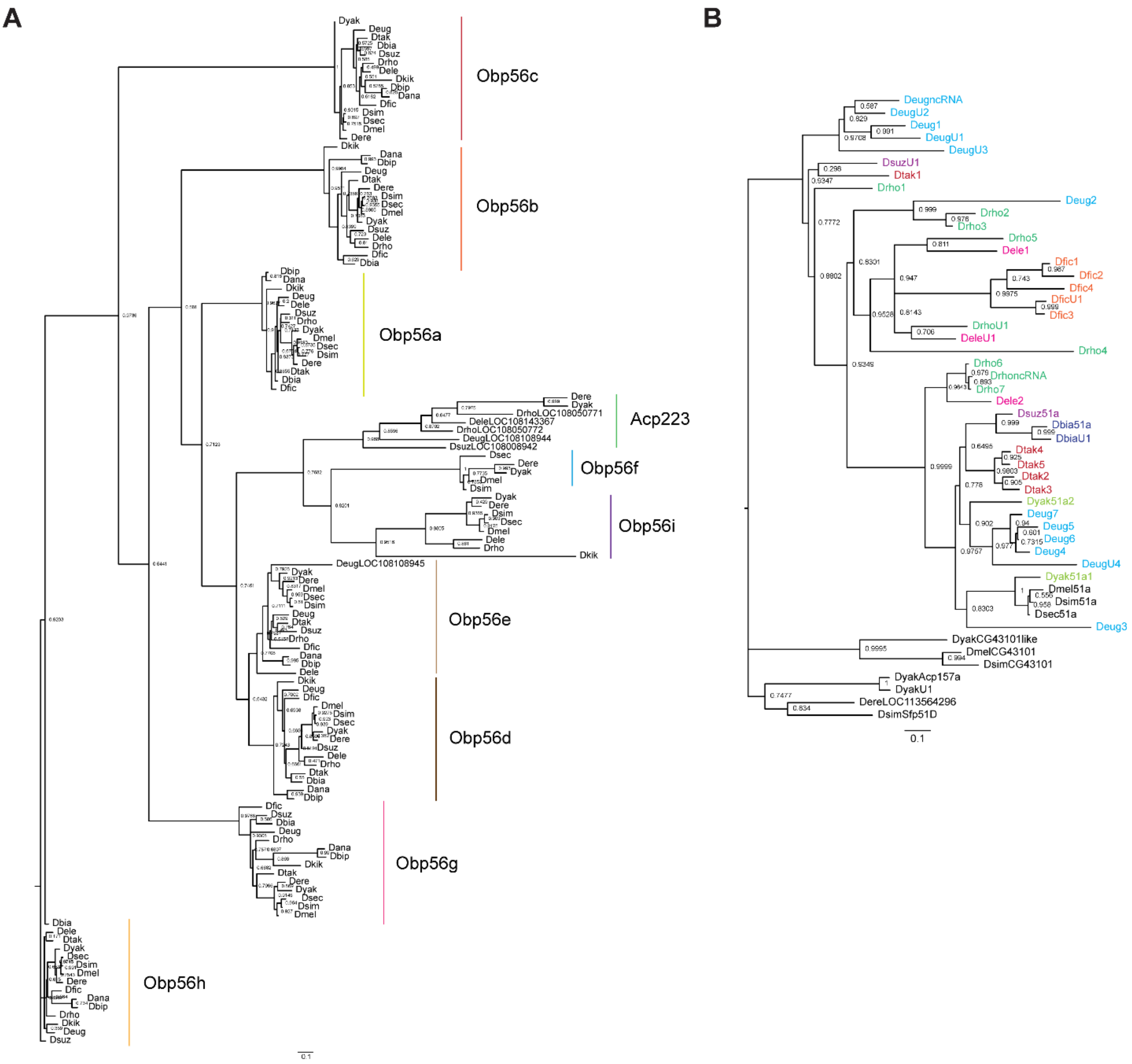

Figure 5—figure supplement 7: RAXML-NG maximum likelihood inferred trees for genes in the A) *Obp56* cluster across *melanogaster* group species, or B) *Obp51a* cluster, where genes are colored as in Figure 5—figure supplement 1. Node values are bootstrap support estimates based on 1,000 replicates. *CG43101* is a gene located next to *Obp51a* in *D. melanogaster*, which has 6 cysteines in a pattern reminiscent of the *Obp* “domain” but is not a predicted *Obp* based on InterProScan searches.

ioRxiv preprint doi: https://doi.org/10.1101/2023.02.03.526941; this version posted February 7, 2023. The copyright holder for this preprint
hich was not certified by peer review) is the author/funder, who has granted bioRxiv a license to display the preprint in perpetuity. It is made
available under aCC-BY 4.0 International license.

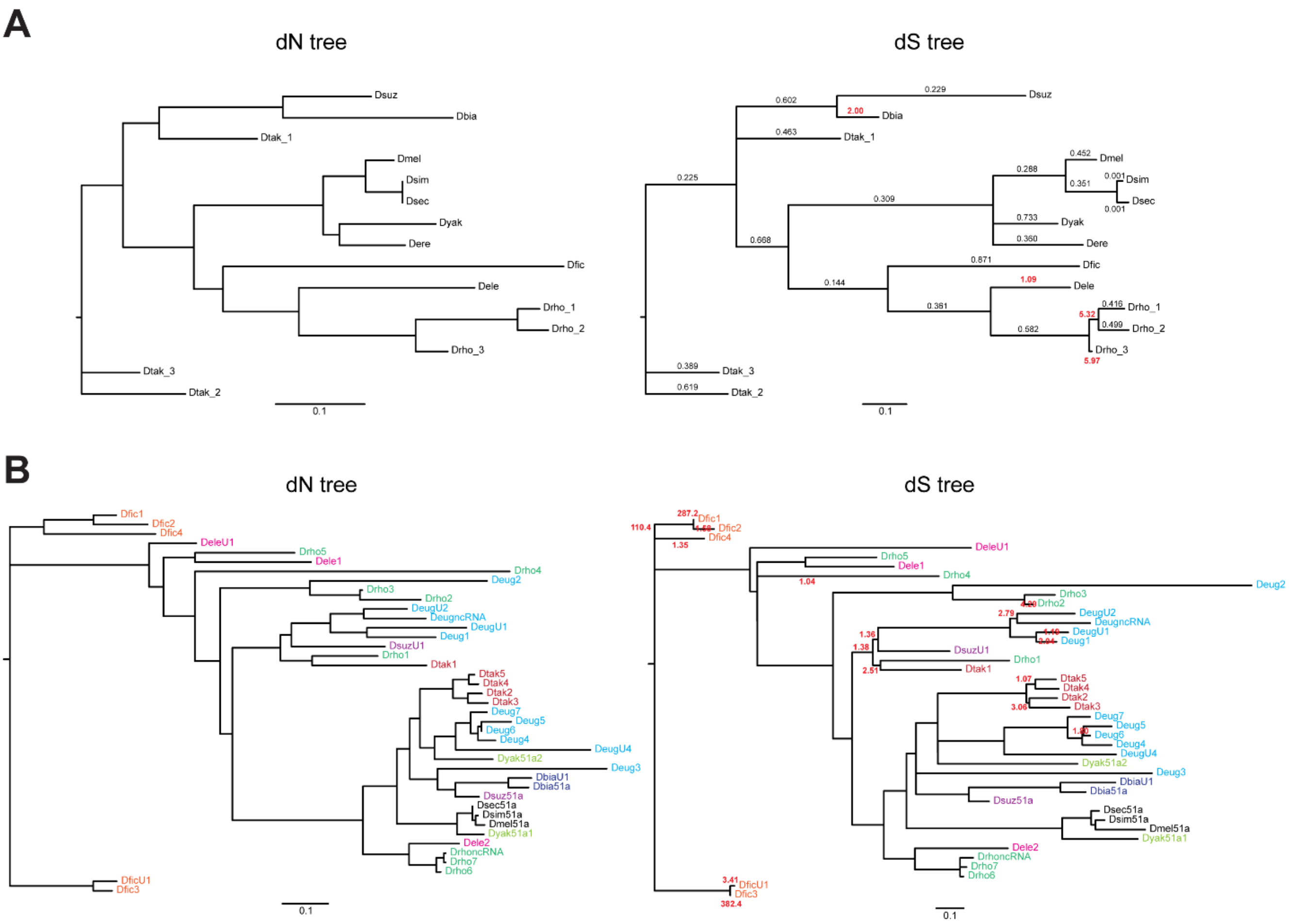

Figure 5—figure supplement 8: A) *Obp22a* and B) *Obp51a* maximum likelihood inferred gene trees, where branch lengths are proportional to either estimates of dN (left) or dS (right) from PAML. Values indicated on the dS tree represent ML-inferred estimates of ω from PAML's free ratio model, where the value is bold and red if ω>1. Values on the *Obp51a* dS tree are only shown if ω >1 for clarity. Genes in B) are color-coded by species if more than one paralog is present in that species' genomes.

bioRxiv preprint doi: https://doi.org/10.1101/2023.02.03.526941; this version posted February 7, 2023. The copyright holder for this preprint (which was not certified by peer review) is the author/funder, who has granted bioRxiv a license to display the preprint in perpetuity. It is made available under aCC-BY 4.0 International license.

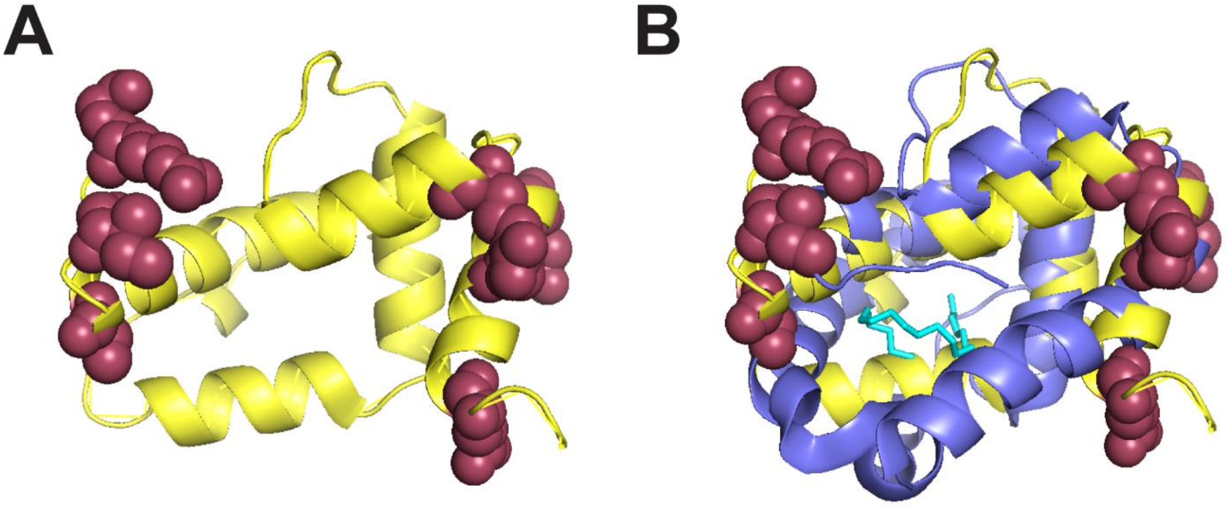

Figure 5—figure supplement 9: Positively selected sites in *Obp22a* cluster on the outward-facing region of the protein. A) Alphafold predicted protein structure (yellow) of Obp22a with the positively selected sites (Pr>0.90 BEB from model M8 of PAML) shown in maroon (Jumper et al., 2021). B) The same structure as A with a superimposed alignment of the crystal structure of Obp76a (LUSH) from (Laughlin et al., 2008), (purple). The cyan molecule represents cVA and the inferred region of the binding pocket.

bioRxiv preprint doi: https://doi.org/10.1101/2023.02.03.526941; this version posted February 7, 2023. The copyright holder for this preprint (which was not certified by peer review) is the author/funder, who has granted bioRxiv a license to display the preprint in perpetuity. It is made available under aCC-BY 4.0 International license.

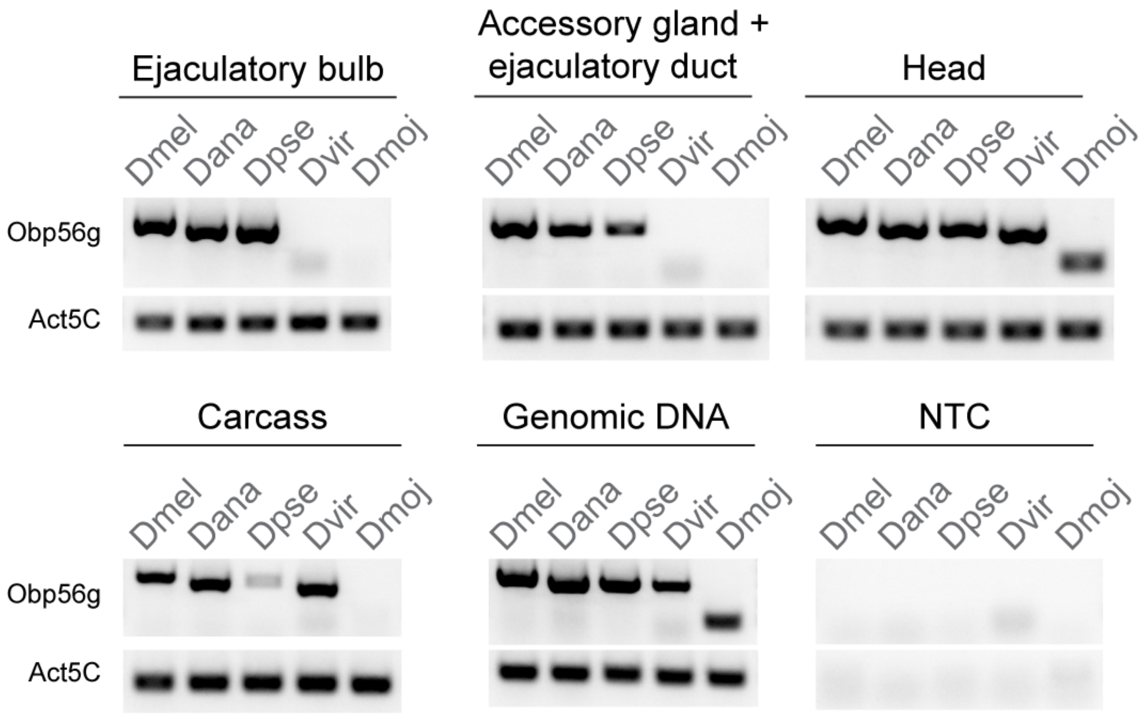

Figure 6—figure supplement 1: Semi-quantitative RT-PCR data from dissected tissues (head, accessory gland + ejaculatory duct, ejaculatory bulb, and carcass) *from D. melanogaster* (Dmel), *D. ananassae* (Dana), *D. pseudoobscura* (Dpse), *D. virilis* (Dvir), and *D. mojavensis* (Dmoj) males after 35 cycles of PCR. NTC = no template control.

Figure 6—figure supplement 1—source data 1: raw and uncropped, labeled gel images for data shown in Figure 6—figure supplement 1.

Supplementary tables:

**Gene**	**gRNA sequence (5'->3')**
Obp8a	1: GGTGAGGATCGCATGGGCAC<br>2: GCTGGACAGGATGCAGTTCG<br>3: ACATGTCCGATGTCATCAAT
Obp22a	1: AATTGTAAGCGAGTGTGCCA<br>2: GAACAATGTTCATAGGAAGA<br>3: AAAGTGAGGGGGATAGATAG

bioRxiv preprint doi: https://doi.org/10.1101/2023.02.03.526941; this version posted February 7, 2023. The copyright holder for this preprint (which was not certified by peer review) is the author/funder, who has granted bioRxiv a license to display the preprint in perpetuity. It is made available under aCC-BY 4.0 International license.

Obp51a	1: TGACAGCTAACAACAGAACC<br>2: GAACGAATGTGCTAAAAAAC<br>3: TAAATTCTCGTTTCAAGCAC
Obp56e	1: TGAGGCTAAGCAGAGAGCCA<br>2: CAAGCTATTGCCCTGCGGTC<br>3: GCCAAGTGTGACTCGACCAA
Obp56f	1: AGCCTGCTTGAAACGGCAGC<br>2: CACTGCTTACTGGAAGTGAA<br>3: ATGTTTAGAAGTCTAATGCT
Obp56g	1: GCAAGCCAACATAGACAGTT<br>2: CGGTGTCACTCCCCAGGATC<br>3: CGGATCGTTAAGACCCTAAT
Obp56i	1: GGTACAAGCAGGTCCCATTA<br>2: CGTCATGAGACCGACGACCC<br>3: CGAAGAACTCGAAATCACAG
ebony (gRNA sequence from Kane et al., 2017)	GCCACAATTGTCGATCGTCA

Table S1: gRNA sequences from flyCRISPR's Optimal Target Finder tool for each *Obp* gene.

**Primer**	**Primer Sequence (5' -> 3')**	
Primer 1F	TTCCCGGCCGATGCAnnnnnnnnnnnnnnnnnnnnGTTTaAGAGCTAtgctgGAAAcag	n: 20nt gRNA 1
Primer 1R	nnnnnnnnnnnnnnnnnnnnTGCACCAGCCGGGAATC	n: 20nt gRNA 2 (RevComp)
Primer 2F	nnnnnnnnnnnnnnnnnnnnGTTTaAGAGCTAtgctgGAAAcag	n: 20nt gRNA 2

bioRxiv preprint doi: https://doi.org/10.1101/2023.02.03.526941; this version posted February 7, 2023. The copyright holder for this preprint (which was not certified by peer review) is the author/funder, who has granted bioRxiv a license to display the preprint in perpetuity. It is made available under aCC-BY 4.0 International license.

Primer 2R	nnnnnnnnnnnnnnnnnnnnTGCACCAGCCGGGAATC	n: 20nt gRNA 3 (RevComp)
Primer 3F	nnnnnnnnnnnnnnnnnnnnGTTTaAGAGCTAtgctgGAAAcag	n: 20nt gRNA 3
Primer 3R	TTCcagcaTAGCTCTtAAACnnnnnnnnnnnnnnnnnnnnTGCACCAGCCGGGAATC	n: 20nt gRNA 4 (RevComp)

Table S2: Primer sequences for cloning gRNAs from Table S1 into pAC-U63-tgRNA-Rev using pMGC as a PCR template (from Poe et al., 2018).

**Gene**	**Primer sequence (5’ -> 3’)**	**Purpose of primer pair**
Obp8a	0F: TCGTAGGTCAGCAGCCCATTAC<br>0R: TCGCATATGACTTTCAATCCGTGT<br>1F: CGTGGGAATGATGCGGAGA<br>1R: CATGGGCAGCATCCTCGAAT	0: Sequencing CRISPR mutants<br>1: RT-PCR
Obp22a	2F: CCACTTTGTATTGGCAACCGCA<br>2R: CAGTCCGCCCAACTTTGAGTTT<br>3F: TGTACTTCTGCTTGGCCTCTC<br>3R: TTTTGGAAGGATTCTGCACAC	2: Sequencing CRISPR mutants<br>3: RT-PCR
Obp51a	4F: AGCAATCTCCCTCACGTGATAT<br>4R: TGCGGCGCTCATGTTTCTTTTA<br>5F: GGCCTGGTTCTGTTGTTAGC<br>5R: TCAAGCACTGGAACACCAAG	4: Sequencing CRISPR mutants<br>5: RT-PCR
Obp56e	6F: ACCTGACAACAAGAAATAACCCGC<br>6R: CACTAGAGCAAGCGTTCCGTTC<br>7F: CCCTTGCAGCTCTATCTTTGG<br>7R: CTTGGTCGAGTCACACTTGG	6: Sequencing CRISPR mutants<br>7: RT-PCR

bioRxiv preprint doi: https://doi.org/10.1101/2023.02.03.526941; this version posted February 7, 2023. The copyright holder for this preprint (which was not certified by peer review) is the author/funder, who has granted bioRxiv a license to display the preprint in perpetuity. It is made available under aCC-BY 4.0 International license.

Obp56f	8F: GGTAACAGTCCCTGGAAACCGA<br>8R: GCGCTTTGCCCGGAATAATCTT<br>9F: TTCATTTTCATCTCTGCTATCTGG<br>9R: GCCCAATTCACATTTTCCTG	8: Sequencing CRISPR mutants<br>9: RT-PCR
Obp56g	10F: GTTAGAAACCTTGACAGTGGCA<br>10R: ATGGGGTAGGCAGTGTATCCCT<br>11F: AGGGCTACATTCGCATTGAC<br>11R: ACCTGTCCAAATCCTTTTCG	10: Sequencing CRISPR mutants<br>11: RT-PCR
Obp56i	12F: ACCTCCATTCGGGTATCTCGAC<br>12R: GACTGAGTGATGCAAAGCACGT<br>13F: TGCTGTGCATTATTGTTAGTCG<br>13R: ACTCGTCATGGGATGTCTCG	12: Sequencing CRISPR mutants<br>13: RT-PCR
Actin 5C	F: AGCGCGGTTACTCTTTCACCAC<br>R: GTGGCCATCTCCTGCTCAAAGT	RT-PCR control gene
D. ananassae Obp56g	F: TGACTCTGCTGCTTAGCTGC<br>R: GATCCTTGTCCACCTGAGCC	
D. pseudoobscura Obp56g	F: GGAGCCGGAGACATAAGCAA<br>R: GCAGGTTTCCTTTCGCATCC	
D. mojavensis Obp56g	F: AGAAGCCCGAAATGACCCAG<br>R: CTCCAGCTTCACCTCACCAG	
D. virilis Obp56g	F: GCTGCTTCTCGGCTGTCTAA<br>R: CCTTAGCTGGCGCATCCTTA	

Table S3: Primer sequences used in this study.

**Gene**	**Allele designation**	**Mutant allele description**
Obp8a	*Obp8a*$^{\Delta 390}$	390 bp deletion in exon 2 between gRNA 1 + 3 (95% of non-signal peptide sequence)

bioRxiv preprint doi: https://doi.org/10.1101/2023.02.03.526941; this version posted February 7, 2023. The copyright holder for this preprint (which was not certified by peer review) is the author/funder, who has granted bioRxiv a license to display the preprint in perpetuity. It is made available under aCC-BY 4.0 International license.

Obp22a	*Obp22a*$^{\Delta 257}$	257 bp deletion in exon 2 between gRNA 1 + 3 (86% of non-signal peptide sequence)
Obp51a	*Obp51a*$^{\Delta 16}$	16 bp deletion in middle of signal peptide region of exon 1 within gRNA 1 site (predicted frameshift and early stop codon)
Obp56e	*Obp56e*$^{\Delta 239}$	239 bp deletion in exon 2 between gRNA 1 + 3 (69% of non-signal peptide sequence)
Obp56f	*Obp56f*$^{\Delta 226}$	226 bp deletion in exon 2 between gRNA 2 + 3 (67% of non-signal peptide sequence + 13 bp into 3' UTR)
Obp56g	*Obp56g*$^{\Delta 333}$	333 bp deletion in exon 2 between gRNA 1 + 3 (95% of non-signal peptide sequence + 7 bp into 3' UTR)
Obp56i	*Obp56i*$^{\Delta 359}$	359 bp deletion in exon 2 between gRNA 1 + 3 (98% of non-signal peptide sequence)

Table S4: CRISPR mutant allele summary for each *Obp* gene.

**Gene**	**4-day receptivity**	**BH-adjusted *p*-value**
Obp8a	KO: 2/17<br>Control: 10/18	0.069
Obp22a	KO: 1/15<br>Control: 0/19	1
Obp51a	KO: 3/19<br>Control: 1/15	1
Obp56e	KO: 0/17<br>Control:1/19	1
Obp56f	KO: 3/18<br>Control: 4/15	1
Obp56i	KO: 1/17<br>Control: 3/14	0.912

bioRxiv preprint doi: https://doi.org/10.1101/2023.02.03.526941; this version posted February 7, 2023. The copyright holder for this preprint (which was not certified by peer review) is the author/funder, who has granted bioRxiv a license to display the preprint in perpetuity. It is made available under aCC-BY 4.0 International license.

Table S5: Four-day receptivity data from an additional replicate using CRISPR mutant males of the genotypes indicated.

**Gene**	**Male genotype**	**% CS females with mating plugs present after copulation**
Obp8a	*Obp8a*$^{\Delta 390}$	100% (n=7)
	Obp8a^{WT}	100% (n=9)
Obp22a	*Obp22a*$^{\Delta 257}$	100% (n=8)
	Obp22a$^{\Delta 257}$*/ CyO*	100% (n=9)
Obp51a	*Obp51a*$^{\Delta 16}$	100% (n=8)
	Obp51a$^{\Delta 16}$*/ CyO*	100% (n=8)
Obp56e	*Obp56e*$^{\Delta 239}$	100% (n=8)
	Obp56e$^{\Delta 239}$*/ CyO*	100% (n=9)
Obp56f	*Obp56f*$^{\Delta 226}$	90% (n=10)
	Obp56f$^{\Delta 226}$*/ CyO*	100% (n=10)
Obp56g	*Obp56g*$^{\Delta 333}$	0% (n=14)
	Obp56g$^{\Delta 333}$*/ CyO*	100% (n=11)
Obp56i	*Obp56i*$^{\Delta 359}$	100% (n=10)
	Obp56i$^{\Delta 359}$*/ CyO*	100% (n=10)

Table S6: Proportion of CS females mated to CRISPR mutant males with morphologically normal mating plugs assessed immediately after the end of mating.

Supplemental methods:

To build our gRNA-expressing vectors, we used pAC-U63-tgRNA-Rev, a plasmid that expresses multiplexed gRNAs separated by rice Gly tRNA sequences, as well as the (F+E)

bioRxiv preprint doi: https://doi.org/10.1101/2023.02.03.526941; this version posted February 7, 2023. The copyright holder for this preprint (which was not certified by peer review) is the author/funder, who has granted bioRxiv a license to display the preprint in perpetuity. It is made available under aCC-BY 4.0 International license.

gRNA scaffold, under the control of the *Drosophila* U6:3 promoter (Poe et al., 2018). We designed Gibson assembly primers containing our gRNA sequences according to (Poe et al., 2018, Table S2). We used these primers to generate PCR products using the pMGC template vector and purified products of the correct size using a gel extraction kit (Poe et al., 2018, Zymo). The empty pAC-U63-tgRNA-Rev plasmid was digested using *Sap*I, and the digested vector and purified PCR products were assembled using the HiFi assembly kit (NEB, NEBuilder). The pAC-U63-tgRNA-Rev and pMGC plasmids were generous gifts from Chun Han at Cornell University.
